# Disability, comorbidities and risk determinants at end of TB treatment in Kenya, Uganda, Zambia and Zimbabwe

**DOI:** 10.5588/ijtldopen.24.0082

**Published:** 2024-05-01

**Authors:** The Kenya, Uganda, Zambia, and Zimbabwe TB Disability Study Group (members listed here in alphabetical order):, S.A. Adakun, F.M. Banda, A. Bloom, M. Bochnowicz, J. Chakaya, A. Chansa, H. Chiguvare, R. Chimzizi, C. Colvin, J.P. Dongo, A. Durena, C. Duri, R. Edmund, A.D. Harries, I. Kathure, F.N. Kavenga, Y. Lin, H. Luzze, I. Mbithi, M. Mputu, A. Mubanga, D. Nair, M. Ngwenya, B. Okotu, P. Owiti, A. Owuor, P. Thekkur, C. Timire, S. Turyahabwe, E. Tweyongyere, M. YaDiul, R. Zachariah, K. Zimba

**Affiliations:** ^1^ Mulago National Referral Hospital, Kampala, Uganda;; ^2^ University Teaching Hospital, Ministry of Health, Lusaka, Zambia;; ^3^ Credence, Contractor for USAID Health Training, Advisory, and Support Contract (GHTASC), Washington DC, USA;; ^4^ Department of Medicine, Therapeutics, Dermatology and Psychiatry Kenyatta University, Nairobi,; ^5^ Respiratory Society of Kenya, Nairobi, Kenya;; ^6^ Ndola Teaching Hospital, Ministry of Health, Ndola, Zambia;; ^7^ United States Agency for International Development (USAID), Harare, Zimbabwe;; ^8^ Ministry of Health, USAID Long-term Exceptional Technical Assistance Project, Genesis, Lusaka, Zambia;; ^9^ International Union Against Tuberculosis and Lung Disease (The Union) Uganda Office, Kampala, Uganda;; ^10^ Directorate of Health Services, Harare City Council, Harare, Zimbabwe;; ^11^ The Union, Paris, France;; ^12^ Department of Clinical Research, Faculty of Infectious and Tropical Diseases, London School of Hygiene & Tropical Medicine, London, UK;; ^13^ Ministry of Health, Division of National TB, Leprosy and Lung Disease Programme, Nairobi, Kenya;; ^14^ Ministry of Health and Child Care, AIDS and TB Department, Harare, Zimbabwe;; ^15^ National Tuberculosis and Leprosy Programme, Ministry of Health, Kampala, Uganda;; ^16^ National Tuberculosis Programme, Ministry of Health/USAID TBLON, Lusaka; Zambia;; ^17^ USAID, Health, Population and Nutrition Office, Kenya and East Africa, Nairobi,; ^18^ Kenyatta National Hospital, Nairobi, Kenya;; ^19^ USAID, Washington DC, USA;; ^20^ UNICEF/UNDP/World Bank/WHO Special Programme for Research and Training in Tropical Diseases (TDR), World Health Organization, Geneva, Switzerland;; ^21^ USAID, Lusaka, Zambia

**Keywords:** TB-associated disability, TB-associated lung disease, Real-time operational research, SORT IT, Universal health coverage

## Abstract

**BACKGROUND:**

We examined the feasibility of assessing and referring adults successfully completing TB treatment for comorbidities, risk determinants and disability in health facilities in Kenya, Uganda, Zambia and Zimbabwe.

**METHODS:**

This was a cross-sectional study within national TB programmes.

**RESULTS:**

Health workers assessed 1,063 patients (78% of eligible) in a median of 22 min [IQR 16–35] and found it useful and feasible to accomplish in addition to other responsibilities. For comorbidities, 476 (44%) had HIV co-infection, 172 (16%) had high blood pressure (newly detected in 124), 43 (4%) had mental health disorders (newly detected in 33) and 36 (3%) had diabetes mellitus. The most common risk determinants were ‘probable alcohol dependence’ (15%) and malnutrition (14%). Disability, defined as walking <400 m in 6 min, was found in 151/882 (17%). Overall, 763 (72%) patients had at least one comorbidity, risk determinant and/or disability. At least two-thirds of eligible patients were referred for care, although 80% of those with disability needed referral outside their original health facility.

**CONCLUSIONS:**

Seven in 10 patients completing TB treatment had at least one comorbidity, risk determinant and/or disability. This emphasises the need for offering early patient-centred care, including pulmonary rehabilitation, to improve quality of life, reduce TB recurrence and increase long-term survival.

## RÉSUMÉ

**CONTEXTE:**

Nous avons examiné la faisabilité d'évaluer et de référer les adultes ayant terminé avec succès le traitement de la TB pour les comorbidités, les déterminants de risque et l'invalidité dans les établissements de santé au Kenya, en Ouganda, en Zambie et au Zimbabwe.

**MÉTHODES:**

Il s'agissait d'une étude transversale menée dans le cadre des programmes nationaux de lutte contre la TB.

**RÉSULTATS:**

Les agents de santé ont évalué 1 063 patients (78% des personnes éligibles) en médiane de 22 min (IQR 16–35) et ont jugé utile et réalisable d'accomplir cette tâche en plus de leurs autres responsabilités. Pour les comorbidités, 476 (44%) étaient co-infectés par le VIH, 172 (16%) souffraient d'hypertension artérielle (dont 124 nouvellement diagnostiqués), 43 (4%) présentaient des troubles de santé mentale (dont 33 nouvellement diagnostiqués) et 36 (3%) étaient diabétiques. Les déterminants de risque les plus courants étaient une « dépendance probable à l'alcool » (15%) et la malnutrition (14%). L'invalidité, définie comme une marche <400 m en 6 min, a été observée chez 151/882 (17%) des patients. Dans l'ensemble, 763 (72%) des patients présentaient au moins une comorbidité, un déterminant de risque et/ou une invalidité. Au moins deux tiers des patients éligibles ont été référés pour des soins, bien que 80% de ceux souffrant d'invalidité aient besoin d'être référés en dehors de leur établissement de santé d'origine.

**CONCLUSIONS:**

Sept patients sur 10 ayant terminé le traitement de la TB présentaient au moins une comorbidité, un déterminant de risque et/ou une invalidité. Cela souligne la nécessité d'offrir des soins précoces centrés sur le patient, y compris une réadaptation pulmonaire, pour améliorer la qualité de vie, réduire la récurrence de la TB et augmenter la survie à long terme.

Globally in 2022, 86% of people newly enrolled on first-line anti-TB treatment successfully completed treatment.^1^ Despite this achievement, a significant proportion (up to 65%) of people who complete TB treatment have post-TB complications that negatively affect their health-related quality of life.^2–5^ People successfully completing TB treatment also have a significantly higher all-cause mortality compared with the general population, with many deaths attributable to non-communicable diseases including cardiovascular disease.^6^ Additionally, a recent study evaluating the lifetime burden of disease due to incident TB found that nearly 50% of the total disability-adjusted life-years (DALYs) was attributed to post-TB sequelae.^7^ Given the current burden of TB-associated disability and mortality, patients with TB merit assessment of their general health status both during and at the end of TB treatment with actions taken to address ongoing illness, risk determinants, comorbidities and disability.^8,9^

Important operational questions, however, about who will carry out these assessments and provide appropriate interventions, and whether this can be routinely done within the healthcare system, need to be addressed.^10,11^ Most national TB programmes (NTPs) currently focus on diagnosis and treatment that is restricted to the duration of TB treatment, as it is often asserted that it is not the responsibility of NTPs and/or they do not have the capacity to take on these additional tasks after treatment is completed.^11^ We believe, however, that there is a case to be made for NTP staff to carry out such assessments at both the start and end of TB treatment. We have outlined the rationale for this previously, highlighting how this could improve TB treatment outcomes, reduce the risk of recurrence, pave the way for pulmonary rehabilitation and address comorbidities, all of which would reduce long-term mortality and improve quality of life.^12^

Such assessments have been carried out in the routine programme setting in China. The first study focused on assessing patients at the end of TB treatment.^13^ The second study focused on conducting assessments both at the start and end of TB treatment, with referrals made to available services for individuals identified with comorbidities, on-going risk determinants and disability.^14^ In both studies, healthcare workers reported that the additional workload was feasible and valuable for their patients. However, further work is needed to determine if it is useful and feasible to carry out similar assessments and referrals in other country contexts, especially at the end of TB treatment. A PubMed search revealed no published studies on this subject from routine programme settings in Africa.

Therefore, the aim of this study was to examine the feasibility and time taken to assess patients successfully completing TB treatment for comorbidities, risk determinants and disability and referring them for care in selected health facilities in Kenya, Uganda, Zambia and Zimbabwe. Specific objectives were to assess 1) the feasibility in terms of proportion of eligible patients who could be assessed, time taken for assessment and proportion of assessments that were found feasible/useful by the HCWs, 2) the proportion with residual symptoms of TB, comorbidities, risk determinants and disability, and 3) the proportion of patients with identified abnormalities referred to routine services for care and support.

## METHODS

### Study design

This was a cross-sectional study carried out within the routine NTP services of four African countries.

### Study sites and setting

The study was conducted in Kenya, Uganda, Zambia and Zimbabwe. The selection of health facilities was guided by logistic convenience and where there were sufficient numbers of TB patients registered each year. Eligible health facilities had to have on-site facilities for blood glucose testing and equipment such as blood pressure measuring machines or easy referral for these tests if equipment was not available or non-functional. TB treatment in all countries was in line with standardized national guidelines. The study was conducted in 26 health facilities; six each in Kenya and Zambia, seven each in Uganda and Zimbabwe. Details of these sites are shown in Supplementary Data S1.

### Study population

The study population included consecutive patients aged ≥18 years who completed TB treatment with any form of TB and assessed at their last follow up visit or at the time of ascertaining their final treatment outcomes. The minimum estimated total sample size was 323 patients, assuming the prevalence of walking <400 m in the 6-min walk test (6MWT) to be 30%,^14^ absolute precision of 5% and 95% confidence level. To maximise precision, the country teams recruited all patients consecutively completing TB treatment from the start of the project until 5 December 2023 (censor date). The recruitment period varied among countries: one month in Kenya, 2 months in Zambia and 4 months each in Uganda and Zimbabwe.

### Training of health professionals through an adapted SORT IT and the development of generic and country-specific study protocols

Training of the study group was carried out using an adapted SORT IT (Structured Operational Research Training Initiative) model focused on implementing real-time implementation research with country teams.^15,16^ From each country, the NTP manager (or suitable representative), the NTP Monitoring and Evaluation Officer, and the appointed in-country study coordinator attended a one-week face-to-face training in March 2023, in Nairobi, Kenya. In this module, participants learnt about key principles of operational research, and how to conduct patient assessments and refer those in need for further care. The teams were also trained on data collection methods using an EpiCollect5 (https://five.epicollect.net/) mobile cloud-based application which allows quality control checks and analysis to be done in real-time. During this module, participants adapted a generic proposal that was developed based on prior experience from two path-finder projects in China.^13,14^ Prior to the face-to-face training, the generic protocol and data collection form (questionnaire) was circulated on-line to the NTP teams of all the four countries. Country specific study protocol and standardized questionnaires were then developed during the one-week face-to-face SORT IT training. The principal investigator and key members of the China study team were also included as part of the Kenya, Uganda, Zambia and Zimbabwe TB disability study group to help with practical guidance. A focal person (doctor, nurse or clinical officer) was appointed in each facility to conduct assessments, refer patients to care and fill a structured data collection form (see Supplementary Data for a sample of the study questionnaire). They were trained by the country representatives who attended the SORT-IT module. At the conclusion of the project, the same study group attended a one-week face-to-face training module in December 2023 in Nairobi, at which they learnt about data analysis and scientific paper writing. At the same time, the study group wrote up this current manuscript.

### Assessment for comorbidities, risk determinants and disability

The focal persons collected demographic and clinical data on all patients, including on-going symptoms at the end of treatment. TB patients were interviewed at either the last follow-up visit or at the time of ascertaining their final treatment outcomes.

### Comorbidities

Comorbidities included HIV infection, diabetes mellitus (DM), high blood pressure and mental health disorders. Patients were asked whether or not they had a known diagnosis of these conditions either prior to starting TB treatment or during TB treatment. For those with no prior diagnosis of DM, a random blood glucose (RBG) followed by a fasting blood glucose (FBG) was performed. An FBG ≥7.0 mmol/L (≥126 mg/dL) or a RBG ≥11.1 mmol/L (≥200 mg/dL) was defined as hyperglycaemia,^17^ with patients referred to a DM clinic for further evaluation of the disease. Patients were screened for high blood pressure, and any person with a systolic blood pressure ≥140 mmHg and/or diastolic blood pressure ≥90 mmHg was diagnosed as having high blood pressure.^18^ Mental health disorder was assessed through the Patient Health Questionnaire 2 (PHQ-2),^19^ and any person with a score ≥3 was referred to a specialist in mental health for confirmation. HIV testing was not performed.

### Risk determinants

Risk determinants included tobacco smoking (smoking anytime in the last 1 month), probable alcohol dependence (Cut, Annoyed, Guilty, Eye questionnaire-CAGE score ≥2) tobacco,^20^ occupational exposure to silica and use of recreational drugs. Malnutrition was defined as body mass index (BMI) <18.5 kg/m^2^. Disability was defined as walking less than 400 m in 6-min walking test (6MWT). The 6MWT is an exercise test used as a proxy for cardio-pulmonary function (aerobic capacity and endurance).^21^ The attending healthcare worker directed patients to walk around a measured track for six minutes.^22^ Details of procedures followed to conduct these assessments at country level are provided in the Supplementary Data.

### Onward referrals for eligible patients

The focal person at each health facility decided whether to offer medical care in the same clinic or refer patients with identified comorbidities, risk determinants and/or disability to other appropriate services in the same health facility or in another institution in the same catchment area. Criteria for referral are shown in Table 1. To where patients were referred are shown in Supplementary Data.

**Table 1.** Referral criteria for further care in TB patients aged ≥18 years who successfully completed TB treatment and were found with comorbidities, risk determinants and/or disability in selected health facilities in Kenya, Uganda, Zambia and Zimbabwe, June–December, 2023.

Condition	Referral criteria
Comorbidities	
HIV infection	Known people living with HIV not on antiretroviral treatment
DM/hyperglycemia	Known diabetes mellitus not in care FBG ≥7 mmol/l in a new person *or* in DM care *OR* RBG ≥11 mmol/l in a new person *or* in DM care
High blood pressure	Known high blood pressure not in care SBP ≥ 140 mmHg *OR* DBP ≥90 mmHg in a new person *or* in care for high blood pressure
Mental health disorder	Known mental health disorder not in care PHQ-2 score ≥3 in a new person or in mental health care
Risk determinants	
Probable alcohol dependence	CAGE score ≥2
Malnutrition	BMI <18.5 kg/m^2^
Occupational exposure to silica	Any occupational exposure to silica
Smoking	Any tobacco smoking in last 1 month
Recreational drug use	Current recreational drug or substance use
Disability	
6MWT	Walked less than 400 m

DM = diabetes mellitus; FBG = fasting blood glucose; RBG = random blood glucose; SBP = systolic blood pressure; DBP = diastolic blood pressure; PHQ-2 = Patient Health Questionnaire 2; CAGE = cut, annoyed, guilty, eye questionnaire; BMI = body mass index; 6MWT = 6 min walk test.

### Data collection, analysis and statistics

Individual patient data were collected using a pre-designed data collection form on EpiCollect5 and cross-checked by each focal person at the implementing sites. The country study coordinator oversaw the work and monitored data quality check reports generated bi-weekly by a data validation team at the Centre for Operational Research (COR), the International Union Against TB and Lung Disease (The Union), Paris, France. Feasibility parameters that were assessed included 1) the overall proportion of patients in the study cohort who could be assessed (response rate), 2) the time taken to perform the assessments, 3) whether the HCWs found it was feasible to conduct these assessments in addition to their usual tasks, and 4) if they felt it was useful for improving the care of TB patients. The questionnaires were administered in English and, where needed, through translation into the local language(s). Data were analysed using STATA^®^ v16.0 (StataCorp, College Station, TX, USA). Continuous data were summarised as means with standard deviations (SDs) or medians with interquartile ranges (IQRs), while categorical data were summarised as frequencies and proportions. Univariate and multivariable binomial regression was performed to assess the characteristics (demographic, clinical, comorbidities and risk determinants) associated with 6MWT < 400 m. The variables with *P* value <0.3 at univariable analysis were included in the multivariable regression. Crude and adjusted prevalence ratios (aPRs) with 95% confidence intervals (CIs) were calculated to assess associations.

Permission to assess TB patients at the end of treatment and use the data was obtained from the NTP of each country. The generic protocol was approved for SORT IT by The Union Ethics Advisory Group, Paris, France (EAG 01/2023 on 13/02/2023). Country specific protocols were approved by Kenya Medical Research Institute Scientific and Ethical Research Unit, Nairobi, Kenya (KEMRI/4761 on 02/10/2023), Mulago Research Ethics Committee, Kampala, Uganda (MHREC2023-96 on 17/07/2023), University of Zambia Biomedical Research Committee, Lusaka, Zambia (4124-2023 on 22/06/2023) and the Medical Research Council of Zimbabwe, Harare, Zimbabwe (MRCZ/A/3018 on 19/05/2023). Informed consent was obtained from all patients recruited for the study.

## RESULTS

### Demographic and clinical characteristics

Of 1355 TB patients successfully completing treatment, 1,063 (78%) were assessed. Demographic and clinical characteristics of these 1,063 patients are shown in Table 2. The mean age was 38 years (SD ±12) and 65% were males. Nearly all patients resided in urban areas. The majority (96%) had pulmonary TB, 70% were bacteriologically confirmed, 91% were newly diagnosed and 2% had drug-resistant TB.

**Table 2.** Demographic and clinical characteristics of TB patients aged ≥18 years who successfully completed TB treatment and were assessed for comorbidities, risk determinants and disability in selected health facilities in four African countries, June–December, 2023.

Characteristic	*n* (%)
Total	1,063
Country of recruitment	
Kenya	126 (12)
Uganda	453 (42)
Zambia	316 (30)
Zimbabwe	168 (16)
Age, years	
18–29	295 (28)
30–44	478 (45)
45–59	235 (22)
≥60	55 (5)
Sex	
Male	691 (65)
Female	372 (35)
Living area	
Urban	1,021 (96)
Rural	42 (4)
Site of TB	
Pulmonary	1,018 (96)
Extrapulmonary	45 (4)
Type of TB	
Bacteriologically confirmed	739 (70)
Clinically diagnosed	324 (30)
Category of TB	
New	971 (91)
Previously treated	92 (9)
Drug susceptibility	
Pan-susceptible	1,044 (98)
RR/MDR-TB	19 (2)
Duration for post-TB assessment, min*	
<15	236 (22)
15–29	470 (44)
30–44	197 (19)
≥45	160 (15)

* Time taken to perform assessments of comorbidities, risk determinants and disability.

RR = rifampicin-resistant; MDR-TB = multidrug-resistant TB.

### Feasibility of conducting assessments

All health workers found it feasible to accomplish the assessments in addition to their other responsibilities and they felt these were useful to improve care for their patients. The median time required to perform assessments was 22 min (IQR 16‒35) with a breakdown as shown in Table 2.

### Ongoing symptoms, comorbidities, risk determinants and disability

Prevalence of on-going symptoms, comorbidities, risk determinants and disability are shown in Table 3. One quarter of patients had on-going symptoms, with cough the most common. For comorbidities, 476 (44%) had HIV infection diagnosed any time before assessment; 36 (3%) had DM/hyperglycaemia with newly detected hyperglycaemia identified in 13; 172 (16%) had high blood pressure with newly detected high blood pressure in 124; and 43 (4%) had a mental health disorder, with newly detected ‘probable depression’ in 33. The two most common risk determinants were ‘probable alcohol dependence’ (15%) and malnutrition (14%). Smoking, occupational exposure to silica dust and use of recreational drugs were found in 5%, 5% and 3% respectively. The 6MWT was carried out in 882 of 895 patients (excluding Zimbabwe), of whom 151 (17%) walked <400 m. The median distance walked in 6 min was 442 m (IQR 410–500). There were 763 (72%) patients who had at least one comorbidity, risk determinant and/or disability at the end of treatment.

**Table 3.** On-going symptoms, comorbidities, risk determinants and disability of TB patients aged ≥18 years who successfully completed TB treatment and were assessed for comorbidities, risk determinants and disability in selected health facilities in Kenya, Uganda, Zambia and Zimbabwe, June‒December, 2023.

Category	*n* (%)
Total	1,063
Symptoms suggestive of TB	
None	784 (74)
Any symptom*	279 (26)
Cough	126 (12)
Shortness of breath	40 (4)
Tiredness/fatigue	46 (4)
Chest pain	51 (5)
Other^†^	97 (9)
Comorbidities	
HIV status	
Positive	471 (44)
Negative	591 (56)
Unknown	1 (<1)
DM/hyperglycaemia
Already known	23 (2)
Tested for DM with either RBG/FBG	962 (93)^‡^
Newly detected hyperglycaemia	13 (1)^§^
Prevalent DM/hyperglycaemia (already known and new)	36 (3)
High blood pressure
Already known	48 (5)
Newly detected	124 (12)^§^
Prevalent high blood pressure (already known and new)	172 (16)
Mental health disorder
Already known	10 (1)
Newly detected, with probable depression	33 (3)^§^
Prevalent mental health disorder (already known and new probable depression)	43 (4)
Risk determinants	
Probable alcohol dependence: CAGE score ≥2	159 (15)
Malnutrition BMI<18.5 kg/m^2^	146 (14)
Occupational exposure: silica dust	52 (5)
Smoked tobacco: anytime in last 1 month	53 (5)
Recreational drug use: current use^¶^	28 (3)
Disability: 6MWT	
Done	882^#^ (99)
6MWT <400 m	151 (17)
Multimorbidity: comorbidity and/or risk determinant and/or disability	
None	300 (28)
One	398 (38)
Two	224 (21)
Three and above	141 (13)

* Some patients had multiple symptoms.

† Included myalgia, joint pain, abdomen discomfort and numbness of hands/feet.

‡ Percentages calculated with those not already known to have DM as denominator.

§ Percentages calculated with those who were not already known to have the condition and assessed for the condition as denominator.

¶ Included marijuana, trihexyphenidyl, *kuber* (chewable nicotine).

# Only 882 patients underwent 6MWT among 895 patients recruited from Kenya, Uganda and Zambia.

DM = diabetes mellitus; RBG = random blood glucose; FBG = fasting blood glucose; CAGE = cut, annoyed, guilty, eye questionnaire; BMI = body mass index; 6MWT = 6 min walk test.

### Characteristics associated with disability (6MWT < 400 m)

Characteristics associated with disability are shown in Table 4. On adjusted analysis, the significant associations were age 45–59 years (aPR 2.2, 95% CI 1.4–3.5), age ≥60 years (aPR 4.8, 95% CI 2.9–7.7) and having a mental health disorder (aPR 2.4, 95% CI 1.3–4.2). There were no significant associations with type of TB, category of TB, drug sensitivity type or smoking.

**Table 4.** Characteristics associated with disability (6MWT <400 m) in TB patients aged ≥18 years who successfully completed TB treatment in selected health facilities in Kenya, Uganda and Zambia, June‒December, 2023.

Variable	Total	6MWT < 400 m	cPR (95% CI)	aPR (95% CI)
	*n*	*n* (%)		
Total	882	151 (17)		
Age, years				
18–29	261	27 (10)	1	1
30–44	398	53 (13)	1.3 (0.8–1.9)	1.2 (0.7–1.8)
45–59	186	50 (27)	2.6 (1.7–3.9)	2.2 (1.4–3.5)
≥60	37	21 (57)	5.5 (3.5–8.6)	4.8 (2.9–7.7)
Sex				
Male	583	94 (16)	1	1
Female	299	57 (19)	1.2 (0.8–1.6)	1.2 (0.9–1.6)
Site of TB				
Pulmonary	852	145 (17)	1	
Extrapulmonary	30	6 (20)	1.2 (0.6–2.4)	
Type of TB				
Bacteriologically confirmed	607	102 (17)	1	
Clinically diagnosed	275	49 (18)	1.1 (0.7–1.4)	
Category of TB				
New	805	137 (17)	1	
Previously treated	77	14 (18)	1.1 (0.6–1.8)	
Drug susceptibility				
Pan-susceptible	864	150 (17)	3.1 (0.4–21.1)	
Resistant	18	1 (6)	1	
HIV status				
Positive	370	79 (21)	1.5 (1.1–2.0)	1.3 (0.9–1.9)
Negative	512	72 (14)	1	1
Diabetes mellitus/hyperglycaemia				
Yes	28	6 (21)	1.2 (0.6–2.6)	
No	854	145 (17)	1	
High blood pressure				
Yes	124	38 (31)	2.1 (1.5–2.8)	1.4 (0.9–2.2)
No	758	113 (15)	1	1
Mental health disorder				
Yes	37	14 (38)	2.3 (1.5–3.6)	2.4 (1.3–4.2)
No	845	137 (16)	1	1
Probable alcohol dependence				
Yes	143	32 (22)	1.4 (0.9–1.9)	1.6 (0.9–2.6)
No	739	119 (16)	1	1
Malnutrition				
Yes	135	24 (18)	1.0 (0.7–1.5)	
No	747	127 (17)	1	
Occupational exposure to silica				
Yes	29	7 (24)	1.4 (0.7–2.8)	
No	853	144 (17)	1	
Smoked tobacco				
Yes	43	4 (9)	0.5 (0.2–1.4)	0.4 (0.1–1.1)
No	839	147 (18)	1	1
Recreational drug use				
Yes	22	3 (14)	0.8 (0.2–2.3)	
No	860	148 (17)	1	
Multimorbidity (excluding 6MWT)				
None	292	29 (10)	1	1
One	350	61 (17)	1.7 (1.2–2.7)	1.2 (0.7–1.9)
Two	165	44 (27)	2.7 (1.7–4.1)	1.2 (0.6–2.5)
Three and above	75	17 (23)	2.3 (1.3–3.9)	1.1 (0.4–3.1)

6MWT = 6 min walk test; cPR = crude prevalence ratio; CI = confidence interval; aPR = adjusted PR.

### Referral for care

The eligibility and referral for further care are shown in Table 5. At least two thirds of all eligible patients were referred for care. More than 79% of those with a comorbidity were referred for care within the same health facility as were almost all those with malnutrition (98%). For other conditions, the proportions of patients provided care within the same health facility were 56% for alcohol dependence, 50% for recreational drug use, 40% for smoking, and 28% for occupational exposure to silica. Of those with 6MWT <400 m, only 20% could be referred within the same health facility (80% needed referral outside their original health facility.

**Table 5.** Status of referral for further care in those identified with comorbidities, risk determinants and disability among TB patients aged ≥18 years who successfully completed TB treatment in selected health facilities in Kenya, Uganda, Zambia and Zimbabwe, June‒December 2023.

Condition	Eligible for referral*	Referred to care	Referred within the same facility
	*N*	*n* (%)	*n* (%)^†^
Comorbidities			
Diabetes mellitus/hyperglycaemia	24	19 (79)	15 (79)
Hypertension	165	154 (93)	125 (81)
HIV	3	2 (67)	2 (100)
Mental health disorder	38	37 (97)	31 (84)
Risk determinants			
Probable alcohol dependence	159	126 (79)	71 (56)
Malnutrition	146	124 (85)	121 (98)
Occupational exposure to silica	52	40 (77)	11 (28)
Smoking	53	42 (79)	17 (40)
Recreational drug use	28	24 (86)	12 (50)
Disability: 6MWT <400 m	151	137 (91)	28 (20)

* For diabetes mellitus, hypertension and mental health disorder, patients who were newly diagnosed and patients who were known to have the condition but not on care or had uncontrolled disease were considered eligible for referral.

† Percentages calculated with total referred for the condition as denominator.

6MWT = 6 min walk test.

## DISCUSSION

This first implementation research study conducted within the NTPs of four African countries shows that one quarter of TB patients had on-going symptoms, around one in five had disability (meaning they were unable to walk 400 m in 6 min) and three quarters had at least one comorbidity, risk determinant and/or disability. A substantial number of individuals were newly detected with high blood pressure; the most common risk determinants were malnutrition and probable alcohol dependence. Referrals for care within the same facility were limited for several risk determinants and especially for disability. These findings highlight the need for assessing and providing integrated services, including pulmonary rehabilitation, for TB patients at the end of TB treatment and if possible, earlier to include all stages of their treatment from initiation through to completion of TB treatment. In terms of feasibility, over three quarters of the cohort were assessed within the routine framework of the NTPs, the assessments were conducted in about 22 minutes and all health workers found it feasible to accomplish these assessments in addition to their other responsibilities. All health workers also felt that these assessments were useful in improving care for their patients.

The study findings are important for several reasons. First, they add justification to the argument that a ‘fourth 90’ be added to the 2014 Stop TB Partnership’s 90-(90)-90 targets to ensure that at least 90% of people with TB have a good health-related quality of life.^23^ Second, the WHO has recently released its first policy brief on addressing TB-associated disability^10^ and our country-level experience can contribute to the ‘how to implement’ this policy in the field. Third, the findings support WHO’s call for a global commitment ‘Rehabilitation 2030’, which recognises rehabilitation as an essential health service for all, necessary for achieving Universal Health Coverage,^24^ as well as the first commitment of the 2023 United Nations High-Level Meeting to strengthen comprehensive care including for TB-associated disability.^25^

There were several strengths of the study. It is operationally relevant as it was conducted within programmatic settings of high TB burden countries. The subject matter responds to an identified implementation research priority. Data were collected in real-time with bi-weekly and rigorous data validation measures. Finally, the conduct and reporting of the study adhered to STROBE (Strengthening The Reporting of Observational Studies in Epidemiology) guidelines.^26^

There were some limitations. Nearly one quarter of TB patients completing treatment were not assessed, the reasons for this were not collected. Children were not included, although they may also experience significant TB-associated disability post treatment.^27^ There were delays in obtaining ethics approvals in some countries resulting in variations across countries in the number of individuals recruited. The study mainly included health facilities in urban areas and therefore may not be nationally representative. The limited project time frame did not allow us to assess parameters such as whether those who were referred did eventually receive care, which services were lacking and whether disability affected the livelihood of individuals. Due to the short time frame of the project, our assessment of feasibility was largely limited to obtaining health worker perceptions’ of whether they found it feasible and useful to accomplish the tasks in addition to their usual responsibilities. More in-depth qualitative assessments on feasibility issues are merited in future research. Finally, we encountered ‘teething’ problems with performing the 6MWT in Zimbabwe resulting in exclusion of data collected from Zimbabwe on this parameter. We plan to bridge these gaps in future research.

Despite these limitations, there are a number of policy and practice implications. First, similar to our prior study in China,^13^ health workers took a median of 22 minutes to perform the assessments and found the work feasible and useful. This is encouraging as it reflects enthusiasm and a ‘perceived need’ for this activity for the benefit of patients, both of which are essential to pave the way towards improving patient-centred care.

Second, three quarters of TB patients had comorbidities, risk determinants and/or disability, with the most prevalent being HIV co-infection, high blood pressure, malnutrition, probable alcohol dependence and disability. Unlike other settings,^28,29^ the prevalence of DM/ hyperglycaemia was low at 3%. Both malnutrition and excess alcohol use can increase the risk of TB recurrence^30,31^ and high blood pressure can increase mortality both during and after TB treatment.^32^ This emphasises the importance of identifying and managing these conditions early to improve treatment success, reduce TB recurrence and improve long-term survival. Third, 17% of our cohort had disability measured by the 6MWT; this was significantly associated with increasing age and the presence of a mental health disorder. This proportion is lower than that found in China,^13,14^ possible reasons including a cohort that was considerably younger and a higher proportion of disabled patients who may have died or been lost to follow up (survival bias) during TB treatment as a result of high HIV co-infection in our setting. Of those with disability, 80% needed referral outside their original health facility. This highlights the need for introducing low-cost pulmonary rehabilitation interventions that are simple enough to be performed by front-line health workers and/or trained TB survivors.^33^ It is also important to note that since the 6MWT was only done at the end of TB treatment, we are unable to determine whether this disability was present prior to, or developed during, TB treatment.

Fourth, in-facility care was excellent (>98%) for HIV co-infection and malnutrition, indicating successful coordination and integration of these services. However, in-facility care was sub-optimal for smoking, probable alcohol dependence, occupational exposure to silica, recreational drug use and disability. Mapping of the services available in the same health facility and identifying priority areas that should be made easily accessible either in-facility or by referral is merited. Finally, the adapted SORT IT training of front-line health workers creates a useful and unique synergy between implementing research studies and strengthening the health system capacity to monitor and deliver services in real-time. This approach can equip healthcare workers to take evidence-based actions using real-time data intelligence, especially during outbreaks and pandemics.^34,35^

In conclusion, in four African countries, a substantial proportion of TB patients who completed TB treatment were identified as having comorbidities, risk determinants and/or disability. These findings serve as a call to improve patient-centred care for individuals with TB to improve treatment outcomes, enhance quality of life, reduce TB recurrence and increase long-term survival.

## Supplementary Material


