# Supplementary material for: Disability, comorbidities and risk determinants at end of TB treatment in Kenya, Uganda, Zambia and Zimbabwe
Source: IJTLD Open. 2024 May 1;1(5):197–205. doi: 10.5588/ijtldopen.24.0082 (PMC11249599; doi:10.5588/ijtldopen.24.0082)
Supplement: Supplementary file 1 [file iutld_ijtld_open_24.0082_supplementarydata1.pdf]

http://dx.doi.org/10.5588/ijtldopen.24.0082

Disability, comorbidities and risk determinants at end of TB treatment in Kenya, Uganda, Zambia and Zimbabwe

Supplementary Data 1: Country specific details of facilities included and procedures followed in the study

1a: Kenya

Description of health facilities

Name of health facility	Level of care	Health care worker cadre involved in assessment
Mbagathi Hospital	Secondary	Clinical Officer, Nurse
Mama Lucy Kibaki Hospital	Secondary	Clinical Officer, Community Health Volunteer
St Mary's Mission Hospital, Langata	Secondary	Clinical Officer
Rhodes Chest Clinic	Primary	Nurse
Riruta Health Centre	Primary	Nurse
Baraka Medical Centre	Primary	Clinical Officer, Nurse

Description of procedure

Height: The height was measured in metres using SECA 213 Stadiometer (SECA, Germany) or Detecto. The patient was asked to stand without shoes with their back against the stand. The patient was asked to look straight and the height marker placed at the tallest position of the head.

Weight: Weight was measured in kilograms in a standing position using a digital scale (SECA 813 flat scale (SECA, Germany) or Detecto) after removal of heavy clothes like jackets and bags.

Blood Pressure: An electronic BP machine (Omron) was used. The patient was asked to rest for at least 5 minutes and not to smoke for at least 30 minutes. The patient was asked to sit on a chair, with their arm at heart level. An appropriate cuff was selected and blood pressure was measured on the right arm. Systolic and diastolic blood pressures were recorded in mmHg. Any patient with systolic blood pressure (SBP) ≥140 mmHg and/or diastolic blood pressure (DBP) ≥90 mmHg was reassessed after a 5-minute rest and the second reading was recorded.

Blood Glucose: All sites were issued with an AccuChek Instant Glucometer. Random blood glucose (RBG) was measured at the clinic during the assessment by the attending clinician. Blood drawn from a finger prick made under sterile conditions was directly placed on the glucostrip connected to the machine and the RBG recorded in mmol/L. Any patient found to have RBG ≥7mmol/l was asked to return to the health facility early the next morning before breakfast for a Fasting Blood Glucose measurement.

6-Minute Walk Test (6MWT): In all the sites, the Health Care Provider (HCP) identified a relatively straight stretch within the facility that was not crowded. The distance along this stretch was measured is meters. The patient was asked to walk back and forth (alone or accompanied by the HCP) at a comfortable pace along this stretch for 6 minutes. The HCP kept a tally of the number of times the patient walked back and forth over 6 minutes and the distance walked by each patient was determined by multiplying this number with the distance of the track. The time walked was measured using a Stopwatch (available in downloadable Apps) or recording the starting and stopping time on the phone.

Referral mechanism

**Referral for**	**Referred to**
HIV	ART clinic within the facility
Diabetes mellitus/ hyperglycemia	Primary facilities: General OPD in same facility<br>Secondary facilities: Diabetes Clinic in the medical OPD in same facility
High blood pressure	Primary facilities: General OPD in same facility<br>Secondary facilities: Hypertension Clinic in the medical OPD in same facility
Mental health disorder	Nearest Psychiatry Unit (outside the facility)
Tobacco smoking	Nearest Psychiatry Unit (outside the facility)
Probable alcohol dependence	Nearest Psychiatry Unit (outside the facility)
Occupational exposure to silica	Kenyatta National Hospital (outside the facility)
Recreational drug use	Nearest Psychiatry Unit (outside the facility)
Malnutrition	Nutritional Unit within the facility
Disability (6MWT<400 metres)	Kenyatta National Hospital (outside the facility)

*Referrals were done using referral slips. ART = antiretroviral therapy; OPD = out-patient department

1b: Uganda

Description of health facilities

Name of health facility	Level of care	Health care worker cadre involved in assessment
Mulago National Referral Hospital	Tertiary	Medical officer, Nurse and Community linkage facilitator
Rubaga Hospital	Tertiary	Nurse, Clinical officer
Naguru Regional Referral Hospital	Tertiary	Nurse, Clinical officer
Kiswa Health Centre III	Primary	Nurse
Kisenyi Health Centre IV	Primary	Nurse
Kawaala Health Centre IV	Primary	Clinical officer, Nurse
Kitebi Health Centre III	Primary	Nurse

Description of procedures

Height: The standing height was measured in metres without shoes using RGZ-120 Height and Weight Scale (Jiangsu Suhong Medical Instruments Co.,Ltd China). The patient was asked to stand without shoes with their back against the stand. The patient was asked to look straight and the height marker was placed at the tallest position of the head.

Weight: Weight was measured in kilograms in a standing position using a digital scale (RGZ-120 Height and Weight Scale, Jiangsu Suhong Medical Instruments Co.,Ltd China) after removal of heavy clothes like jackets and bags.

Blood Pressure: The patient was seated, rested for five minutes with arms rested on a surface at the same level of the measuring device. The blood pressure taken on the left arm using the Edan digital machine. Any patient with systolic blood pressure (SBP) ≥140 mmHg and/or diastolic blood pressure (DBP) ≥90 mmHg was reassessed after a 5-minute rest, and the second reading was recorded. BP machines were procured and provided to five facilities as part of the study.

Blood Glucose: The finger of the participant was cleaned and a blood sample obtained by pricking the finger with a sterile lancet. A drop of blood was obtained and placed on the glucostrip of the glucometer (OneTouch Select Simple or Contour Plus). Any patient found to have a random blood glucose (RBG)≥7mmol/l was requested to return to the health facility early the next morning before breakfast for a fasting blood glucose (FBG). Glucometers were procured and provided to three facilities as part of the study. The glucostrips were procured and provided to all facilities as part of the study.

6-minute walk test (6MWT): In all the sites, a straight non-crowded space was identified and the length measured in metres (see table below). The participant was instructed to walk back and forth along the measured track for 6 minutes and the distance covered recorded in metres. The time walked was measured using a Stopwatch on a mobile phone. At the end of the 6 minutes, the distance walked by the participant was measured by multiplying the number of times the patient walked a complete 30 meters. If the distance at the end of 6 minutes was less than 30 meters, then the exact distance was measured by a tape measure, and added to the total of the completed 30 meters distance.

Health Facility	Measured track (in metres)
Kisenyi Health Centre IV	20
Kitebi Health Centre III	30
Naguru Regional Referral Hospital	30

Rubaga Hospital	40
Kiswa Health Centre III	40
Kawaala Health Centre III	35
Mulago National Referral hospital	30

Referral mechanism

**Referral for**	**Referred to**
HIV	ART Clinic within facility
Diabetes mellitus/ hyperglycemia	Tertiary facilities: Diabetes Clinic within the facility<br>Primary facilities: General Medical OPD within the facility
High blood pressure	Tertiary facilities: Hypertension Clinic within the facility<br>Primary facilities: General Medical OPD within the facility
Mental health disorder	National Psychiatric Hospital (Butabika), outside the facilities
Tobacco smoking	National Psychiatric Hospital (Butabika), outside the facilities
Probable alcohol dependence	National Psychiatric Hospital (Butabika), outside the facilities
Occupational exposure to silica	Mulago Lung Institute Clinic, outside the facilities
Recreational drug use	National Psychiatric Hospital (Butabika), outside the facilities
Malnutrition	Nutritional Clinic within facility
Disability (6MWT<400 metres)	Mulago Lung Institute Clinic, outside the facilities

*Referrals were done using referral slips; ART = antiretroviral therapy; OPD = out-patient department

1c: Zambia

Description of health facilities

Name of health facility	Level of care	Health care worker cadre involved in assessment
University Teaching Hospital (UTH)	Tertiary	Nurse, Medical officer
Chipata First Level	Secondary	Nurse
Matero First Level	Secondary	Nurse, Clinical officer
Chipata First Level	Secondary	Nurse, Clinical officer
Chawama First Level	Secondary	Nurse, Clinical officer
Chilenje First Level	Secondary	Nurse

Description of procedures

Height: The standing height was measured in metres using SECA 213 Stadiometer (SECA, Germany). The patients was asked to stand without shoes with their back against the stand. The patient was asked to look straight and the height marker placed at the tallest position of their head.

Weight: Weight was measured in kilograms in a standing position using an analogue scale (Prestige, Hardik Medi Tek, India) after removal of heavy clothes like jackets and bags.

Blood Pressure: Arm blood pressure was measured in mmHg with the patient sitting comfortably on a chair with the back supported using an automatic digital equipment (CITIZEN CH 432, Citizen, Japan). Any patient with a systolic blood pressure (SBP) ≥140 mmHg and/or diastolic blood pressure (DBP) ≥90 mmHg was reassessed after a 5-minute rest, and the second reading was recorded.

Blood glucose: Random blood glucose (RBG) was measured by finger prick using ACCUCHECK by Roche. Any patient with RBG ≥7 mmol/L was requested to come the following morning for fasting blood glucose (FBG) measurement. Glucostrips were procured and provided to all facilities as part of the study.

6-minute walk test (6MWT): Using a tape, a 30-meters distance was measured between two points in a straight line. The patient was instructed on the 6MWT and its objective. The patient was then requested to walk from one point to the next point and to continue walking to-and-fro for 6 minutes. A timer using a watch, or a mobile phone, was used to measure the 6 minutes. At the end of the 6 minutes, the distance walked by the patient was measured by multiplying the number of times the patient walked a complete 30 meters. If the distance at the end of 6 minutes was less than 30 meters, then the exact distance was measured by a tape measure, and added to the total of the completed 30 meters distance.

Referral mechanism

Referral for	Referred to
HIV	ART clinic within the facility

Diabetes mellitus/ hyperglycemia	UTH: Endocrinology Unit of UTH<br>Other facilities: General Medical Clinic in same facility
High blood pressure	UTH: Cardiology Unit<br>Other facilities: General Medical Clinic in same facility
Mental health disorder	UTH: Psychiatry Unit<br>Other facilities: Mental health practitioner in the same facility
Tobacco smoking	UTH: Psychiatry Unit<br>Other facilities: Mental health practitioner in the same facility
Probable alcohol dependence	UTH: Psychiatry Unit<br>Other facilities: Mental health practitioner in the same facility
Occupational exposure to silica	UTH: Respiratory Unit at UTH<br>Other facilities: Respiratory Unit at UTH (outside facility)
Recreational drug use	UTH: Psychiatry Unit<br>Other facilities: Mental health practitioner in the same facility
Malnutrition	Nutritional Unit within the facility
Disability (6MWT<400 metres)	UTH: Respiratory Unit at UTH<br>Other facilities: Respiratory Unit at UTH (outside facility)

*Referrals were done using referral slips; ART = antiretroviral therapy

1d: Zimbabwe

Description of health facilities

Name of health facility	Level of care	Health care worker cadre involved in assessment
Mabvuku Polyclinic	Primary	Nurse, Environmental Health Technician (EHT)
Rujeko Polyclinic	Primary	Nurse, EHT
Hopely Polyclinic	Primary	Nurse, EHT
Glenview Polyclinic	Primary	Nurse, EHT
Kuwadzana Polyclinic	Primary	Nurse, EHT
Mbare Polyclinic	Primary	Nurse, EHT
Beatrice Road Infectious Disease Hospital	Secondary	Nurse, EHT

Description of procedures:

Height: All health facilities used a moveable height board to measure the patient's height. The patient was asked to stand with their back against the board, which was fixed next to a vertical wall. The patient was asked to look straight and the height marker was placed at the tallest position of the head. The height was recorded in centimetres.

Weight: Weight was measured in kilograms using an electronic weight scale (SECA 813) after removing shoes and heavy clothing and any heavy belongings.

Blood Pressure: An electronic BP machine (Omron or AND Medical) was used. The patient was asked to rest for at least 5 minutes and not to smoke for at least 30 minutes. The patient was asked to sit on a chair, with their arm at heart level. An appropriate cuff was selected and the blood pressure was measured on the right arm. Systolic and diastolic blood pressure was recorded in mmHg. Any patient with systolic blood pressure (SBP) ≥140 mmHg and/or diastolic blood pressure (DBP) ≥90 mmHg was reassessed after a 5-minute rest, and the second reading was recorded. BP machines were procured and provided to five facilities as part of the study.

Blood Glucose: All sites used a capillary blood sample that was tested on a glucose test strip (Codefree) for random blood glucose (RBG). The patient's finger was swabbed using an alcohol swab, then pricked using a lancet. Blood drawn was directly placed on the glucostrip connected to a machine and the RBG was recorded in mmol/L. Any patient with RBG ≥7 mmol/L was requested to return the following morning for fasting blood glucose (FBG) measurement. Glucometer machines were procured as part of the study and distributed to six health facilities.

Referral mechanism

Referral for	Referred to
HIV	OI/ART department within the facility

Diabetes mellitus/ hyperglycemia	Tertiary facilities (Sally Mugabe Central Hospital or Parirenyatwa Group of Hospitals), outside the facility
High blood pressure	Tertiary facilities (Sally Mugabe Central Hospital or Parirenyatwa Group of Hospitals), outside the facility
Mental health disorder	Friendship Bench in the same facility
Tobacco smoking	Friendship Bench in the same facility
Probable alcohol dependence	Friendship Bench in the same facility
Occupational exposure to silica	Tertiary facilities (Sally Mugabe Central Hospital or Parirenyatwa Group of Hospitals), outside the facility
Recreational drug use	Friendship Bench in the same facility
Malnutrition	Nutritional Unit in the same facility
Disability (6MWT<400 metres)	Tertiary facilities (Sally Mugabe Central Hospital or Parirenyatwa Group of Hospitals), outside the facility

*Referrals were done using referral slips; OI- opportunistic infection; ART= antiretroviral therapy

Supplementary Data 2: Sample of the data collection proforma at end of anti-TB treatment

Country: ZAMBIA Name of the TB Centre: Form number:	
**I. INITIAL ASSESSMENT**	
TB Registration Number	
Phone number of patient	
Phone number of next of kin	
Date of registration for TB treatment	
A. Age (in completed years)	
B. Sex	☐ Male ☐ Female ☐ Others
C. Living community	☐ Urban ☐ Rural
D. Diagnosis	☐ Pulmonary TB ☐ Extra pulmonary TB
E. Type of TB	☐ Bacteriologically confirmed ☐ Clinically diagnosed
F. Category of TB	☐ New ☐ Re-treatment
G. Drug-susceptibility	☐ DS ☐ INH-R ☐RR ☐MDR ☐Pre-XDR/XDR
**Date of Assessment (dd/mm/yy)**	
**Start time of assessment (HH:MM)**	
**SYMPTOMS, COMORBIDITIES, DETERMINANTS**	
~~H.~~ On a scale of 1 to 5 where 1 is very bad and 5 is very good, how would you rate your overall health today?	☐ 1 ☐ 2 ☐ 3 ☐ 4 ☐ 5
I. Do you have any symptoms? (Tick all that apply)	☐ No symptoms ☐ Cough ☐ Shortness of breath<br>☐ Tiredness/Fatigue ☐ Chest pain<br>☐ Others (Specify):
J. What is your HIV-status?	☐ HIV-positive; ☐ HIV-negative; ☐ Unknown
K. If HIV positive, are you currently in HIV care at ART centre?	☐ Yes ☐ No
L. Do you have Diabetes Mellitus (DM)?	☐ Yes ☐ No ☐ Don't Know
M. If known DM, are you availing care for DM from a health facility?	☐ Yes ☐ No
N. Do you have chronic kidney disease (CKD)?	☐ Yes ☐ No ☐ Don't Know
O. If known CKD, are you availing care for CKD from a health facility?	☐ Yes ☐ No
P. Do you have Hypertension (HTN)?	☐ Yes ☐ No ☐ Don't Know
Q. If known HTN, are you availing care for HTN from a health facility?	☐ Yes ☐ No
R. Do you have a diagnosed mental health (MH) issue?	☐ Yes ☐ No ☐ Prefer not to answer
S. If known MH, what is the mental health issue?	☐ Depression ☐ Anxiety<br>☐ Others (Specify):_____________
T. If known MH, are you availing care for MH from a health facility?	☐ Yes ☐ No
U. Over the last 2 weeks, how often have you been bothered by the following problems (a and b)?	
a) Little interest or pleasure in doing things	☐Not at all-0 ☐Several days-1 ☐More than half the days-2 ☐Nearly every day-3
b) Feeling down, depressed or hopeless	☐Not at all-0 ☐Several days-1 ☐More than half the days-2 ☐Nearly every day-3
c) Whether PHQ2 score is 3 or higher (Ua+Ub)?	☐ Yes ☐ No
V. Whether you smoked tobacco anytime in last one month?	☐ Yes ☐ No
W. If yes how many cigarettes per day?	
X. How often did you have a drink containing alcohol in the last one year?	☐ Never ☐ Monthly or less ☐ 2 to 4 times a month<br>☐ 2 to 3 times a week ☐ 4 or more times a week
Please fill the CAGE(a to e) questions if the response is not 'Never' for X	
a) Have you ever felt you should cut down on your drinking?	☐ Yes -1 ☐ No -0
b) Have people annoyed you by criticizing your drinking?	☐ Yes -1 ☐ No -0

c) Have you ever felt bad or guilty about your drinking?	☐ Yes -1 ☐ No -0
d) Have you ever had a drink first thing in the morning to steady your nerves or to get rid of a hangover (eye-opener)?	☐ Yes -1 ☐ No -0
e) Whether CAGE score is 2 or higher (Xa+Xb+Xc+Xd)	☐ Yes ☐ No
Y. Do you have occupational exposure to silica dust?	☐ Yes ☐ No
Z. Do you use recreational drugs?	☐ Yes ☐ No
AA. If Yes to X, please specify the names of the drug (s)?	
**PHYSICAL EXAMINATION**	
1. Height (in meters)	
2. Weight (in kg)	
3. Is BMI less than 18.5 kg/m^2? (BMI= Weight/Height^2)	☐ Yes ☐ No
4. Systolic Blood pressure (SBP in mm Hg)	
5. Diastolic Blood Pressure (DBP in mm Hg)	
6. Is Blood Pressure elevated (SBP≥140 OR DBP≥90)	☐ Yes ☐ No
7. 6-MINUTE WALK TEST (6MWT)	
a) Test done	☐ Yes ☐ No
b) If No, reason for not being able to do the test	
c) Meters walked in 6-minutes	
d) Was the 6MWT less than 400 meters?	☐ Yes ☐ No
**8. Time at the end of physical examination (HH:MM)**	
**INVESTIGATIONS**	
9. Whether Random Blood Glucose (RBG) done?	☐ Yes, at assessment station ☐ Yes, at lab ☐ No
10. Random Blood Glucose (RBG) level (mmol/l)	
11. Whether RBG≥7.0 mmol/l?	☐ Yes ☐ No
12. If RBG≥7.0 mmol/l, whether Fasting Blood Glucose (FBG) done?	☐ Yes, at assessment station ☐ Yes, at lab ☐ No
13. Fasting Blood Glucose (FBG) level (mmol/l)	
14. Whether FBG≥7.0 mmol/l?	☐ Yes ☐ No
15. Whether serum creatinine was done?	☐ Yes ☐ No
16. Serum Creatinine level (in ug/l)	
17. Whether serum creatinine >120 ug/l?	☐ Yes ☐ No
**DURATION AND FEASIBILITY**	
**18. End time including time taken to do random blood glucose (HH:MM)** *Exclude time taken for serum creatinine test*	
**HEALTH CARE PROVIDER INFORMATION**	
19. What is your job title?	☐ Nurse ☐ Doctor ☐ Physician assistant ☐ Clinical officer ☐ Others(specify)__________________
20. As a care provider, do you feel the assessment was feasible?	☐ Yes ☐ No
21. If No, reason?	
22. As a care provider, do you feel the assessment was useful ?	☐ Yes ☐ No
23. If No, reason?	

Form Number: **II. Referral to care and support**

**Condition**	**A. Requires referral**	**B. If Yes for A, Whether Referred?**	**C. If referred, was referral within the same facility?**	**D. If No for B, Reason***	**Telephonic follow-up after one month of referral**	
					**E. If referred, started care?**	**F. If No for E, Reason***
For HIV care	□Yes □No	□Yes □No	□Yes □No		□Yes □No	
For Diabetes Mellitus care	□Yes □No	□Yes □No	□Yes □No		□Yes □No	
For Hypertension care	□Yes □No	□Yes □No	□Yes □No		□Yes □No	
For CKD	□Yes □No	□Yes □No	□Yes □No		□Yes □No	
For mental health care	□Yes □No	□Yes □No	□Yes □No		□Yes □No	
For quitting smoking	□Yes □No	□Yes □No	□Yes □No		□Yes □No	
For reducing alcohol	□Yes □No	□Yes □No	□Yes □No		□Yes □No	
For exposure to silica	□Yes □No	□Yes □No	□Yes □No		□Yes □No	
For drug dependency	□Yes □No	□Yes □No	□Yes □No		□Yes □No	
For nutritional support	□Yes □No	□Yes □No	□Yes □No		□Yes □No	
For Pulmonary Rehabilitation	□Yes □No	□Yes □No	□Yes □No		□Yes □No	

*** a) Service not available in the facility or nearby facility; b) Patient not willing; c) Others-specify**
